# Fabrication of a Molecularly Imprinted Nano-Interface-Based Electrochemical Biosensor for the Detection of CagA Virulence Factors of *H. pylori*

**DOI:** 10.3390/bios12121066

**Published:** 2022-11-23

**Authors:** Kirti Saxena, Bayu Tri Murti, Po-Kang Yang, Bansi Dhar Malhotra, Nidhi Chauhan, Utkarsh Jain

**Affiliations:** 1 Amity Institute of Nanotechnology, Amity University Uttar Pradesh (AUUP), Sector-125, Noida 201313, India; 2 Graduate Institute of Biomedical Materials and Tissue Engineering, Taipei Medical University, Taipei 11031, Taiwan; 3 Department of Biomedical Sciences and Engineering, National Central University, Chung-li 32001, Taiwan; 4 Nanobioelectronics Laboratory, Department of Biotechnology, Delhi Technological University, Delhi 110042, India

**Keywords:** *H. pylori*, CagA, biosensors, molecularly imprinted polymers, molecular dynamics simulations, electrochemical

## Abstract

*H. pylori* is responsible for several stomach-related diseases including gastric cancer. The main virulence factor responsible for its establishment in human gastric cells is known as CagA. Therefore, in this study, we have fabricated a highly sensitive MIP-based electrochemical biosensor for the detection of CagA. For this, an rGO and gold-coated, screen-printed electrode sensing platform was designed to provide a surface for the immobilization of a CagA-specific, molecularly imprinted polymer; then it was characterized electrochemically. Interestingly, molecular dynamics simulations were studied to optimize the MIP prepolymerization system, resulting in a well-matched, optimized molar ratio within the experiment. A low binding energy upon template removal indicates the capability of MIP to recognize the CagA antigen through a strong binding affinity. Under the optimized electrochemical experimental conditions, the fabricated CagA-MIP/Au/rGO@SPE sensor exhibited high sensitivity (0.275 µA ng^−1^ mL^−1^) and a very low limit of detection (0.05 ng mL^−1^) in a linear range of 0.05–50 ng mL^−1^. The influence of other possible interferents in analytical response has also been observed with the successful determination of the CagA antigen.

## 1. Introduction

Gastric cancer is the fifth most common and prevalent type of cancer that affects older males especially [1,2]. The incidence of gastric cancer mostly depends upon food habits and *Helicobacter pylori* (*H. pylori*) [3]. *H. pylori* infection is the main factor in 70% of cases of gastric cancer. It is a spiral-shaped, microaerophilic gram-negative bacteria that is responsible for various stomach-related diseases such as gastritis, peptic ulcers, and gastric cancer in advanced cases [4,5,6]. Many virulence factors of *H. pylori* are responsible for the establishment of infection in gastric cells or tissues [7,8,9]. CagA or cytotoxin-associated gene A is the most studied toxin of *H. pylori*, which is 120–145 KDa. CagA toxin is known to disrupt the normal differentiation of gastric epithelial cells that include cell polarity, cell adhesion, and cell migration inhibition [10,11,12]. Due to its role in the development of gastric cancer, it is classified as an oncogenic protein of *H. pylori* bacteria. CagA is also utilized as a biomarker in several studies for the successful detection of *H. pylori* [13]. Therefore, as a considerable biomarker, CagA is utilized herein for the development of a biosensing surface for the detection of *H. pylori*. However, conventional methods for *H. pylori* detection are available but they have several disadvantages such as low sensitivity, high cost, a requirement for skilled professionals, and painfully invasive methods (i.e., endoscopy) [14]. In order to overcome these limitations, biosensors show remarkable advantages over conventional methods as they are quick, easy to handle, highly sensitive, and low-cost [15,16]. Biosensors of different types are characterized based on their transducer surface and biorecognition element. Natural receptors such as antigens/antibodies, enzymes, cells, DNA/RNA, aptamers, and peptides are used as biorecognition elements [17,18,19]. They exhibit a very high affinity for their target analyte but also have some challenges in laboratory applications such as poor durability, high-temperature sensitivity, and low stability in high/low pH solutions. In this context, imprinted polymers have been reported as robust artificial receptors/biomimetic materials. They have great potential to overcome the above-mentioned limitations of natural receptors for the recognition of desired analytes. The molecular imprinting technique used the stereochemistry of the targeted analyte for specific molecular recognition in a simple and highly effective manner. Molecularly imprinted polymers, or MIPs, are chemically and physically stable and able to form specific cavities in polymer matrices corresponding to binding analytes. The synthesis process involved the printing of a macromolecular structure onto the polymeric material’s surface using a template molecule to create similar sites to the analyte. The basic synthesis principle of MIP is based on a process in which the copolymerization of functional monomers and cross-linked polymers takes place in the presence of a target analyte. Furthermore, the removal of the template from the polymer matrix after the polymerization process creates specific imprinted cavities (recognition sites) complementary in shape, size, and functionality to the template molecules. Thus, MIPs are attractive to researchers for the development of biosensors for the detection of several infectious diseases [20]. MIPs can be categorized into non-covalent and covalent imprinting on the basis of imprinting strategy. However, the fabrication of MIPs involved various methods; among them, the electropolymerization technique provides easy adherence of the polymeric film on the electrode surface, thus enabling it to control the film thickness of any size and shape. The electropolymerization technique is also a simple and easy-to-handle method [21].

Nanomaterials have also played an important role in the fabrication of electrochemical-based sensing platforms [22,23]. The best-known nanomaterials, reduced graphene oxide (rGO) and gold (Au), were employed in this study to amplify the signals on the electrode surface. rGO and Au nanomaterials provide high conductivity, large surface area for immobilization of biomolecules, and facilitate electron transfer on an electrode [24,25]. Therefore, in the present work, a screen-printed carbon electrode coated with rGO and gold nanoparticles was utilized as a matrix for the fabrication of a CagA detection platform [26,27]. For this, CagA-specific MIP was immobilized on an Au/rGO/SPE surface, which provides a biocompatible and electron-conductive platform. The molecular dynamics (MD) simulations were carried out to predict the optimized molar ratio of the MIP prepolymerization along with their possible modes of interaction. Density functional theory (DFT) calculations were also incorporated to assess the electronic properties and the active sites of the MIP component. Upon MD simulations, the binding energies were calculated, emphasizing the excellent functionality of the MIP-based electrodes in the as-prepared electrochemical biosensor.

## 2. Materials and Methods

### 2.1. Chemical Reagents Used

CagA antigen and antibodies were procured from the Max von Pettenkofer Institute of Hygiene and Medical Microbiology, Ludwig Maximilian University, Munich, Germany and stored at −20 °C till further use. The monomer, cross-linker, initiator, and methanol were purchased from Sigma-Aldrich, India, and were used without any further purification. Other chemicals, including K_3_[Fe(CN)_6_], K_4_[Fe(CN)_6_], and KCl, were purchased from Sisco Research Laboratory (SRL), Mumbai, India. All other chemicals were of analytical reagent (AR) grade and all aqueous solutions were prepared in double distilled water throughout the experiments. Cyclic voltammetric (CV) and electrochemical impedance studies (EIS) were performed using Bio-Logic Science Instruments SP-200 with EC-Lab^®^ software with an impedance analysis module. The working electrode was the screen-printed carbon electrode (SPE). Scanning electron microscopy (SEM) and Brunauer–Emmett–Teller (BET) were used to study the surface morphology and surface area of synthesized MIP with and without template CagA. The Material Studio version 2018r1 software package (Accelrys Inc., San Diego, CA, USA) [28] was used to conduct computational simulations.

### 2.2. Molecular Dynamics Simulations

**Figure 1:**
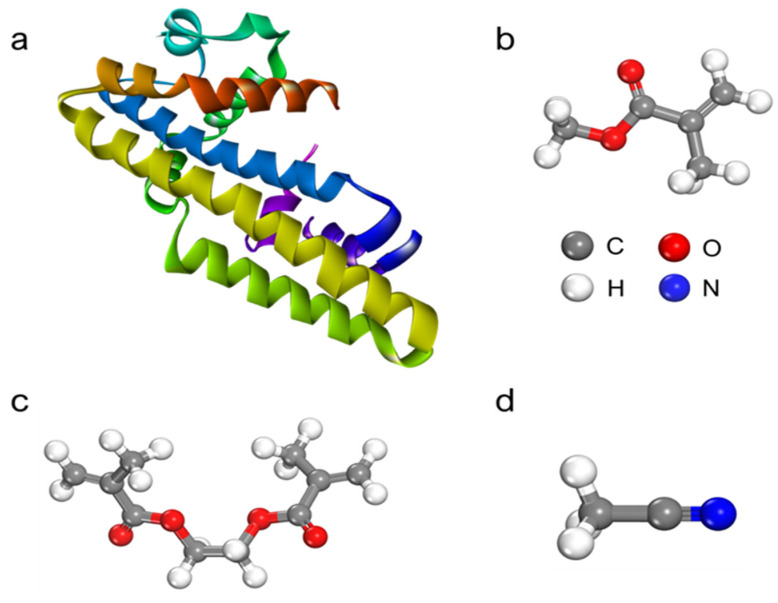
Chemical structures of MIP optimization components including (**a**) CagA, (**b**) MMA, (**c**) EGDMA, and (**d**) ACN. CagA was represented in the ribbon diagram, while other molecules were displayed in the ball and stick model.

All the preparation and geometry optimization of the bulk molecules during MIP prepolymerization were conducted using Material Studio version 2018r1 software package (Accelrys Inc., San Diego, CA, USA). To accurately mimic the experimental condition, the primary virulence determinant of *H. pylori* (i.e., CagA (PDB ID: 4IRV) [29]) was used as the template for MIP, while methyl methacrylate (MMA) served as the functional monomer of the polymerization process (Figure 1a,b). Ethylene glycol dimethacrylate (EGDMA) and acetonitrile (ACN) were employed as the cross-linker and the porogen, respectively (Figure 1c,d). For the small organic molecules (i.e., MMA, EGDMA, ACN), the molecular geometries were optimized using spin-restricted DFT described in the DMol3 code [30,31] with the B3LYP hybrid functional [32,33]. To evaluate the electronic properties of the studied molecules, a double numerical atomic orbital (DNP) basis set with a 3.5 basis file was used along with 3.7 Å of global orbital cut-off. The optimum energy, gradient, and displacement convergences were set to be 1 × 10^−5^ Ha, 2 × 10^−3^ Ha/Å, and 5 × 10^−3^ Å, respectively. Instead, the density functional-based tight binding (DFTB+) [34] method was considered to optimize the MIP template due to the macromolecular structure of the CagA protein which contains thousands of atoms. Non-self-consistent charge (SCC) DFTB was used herein to achieve the convergence during optimization along with the 3rd order parametrization for organic and biological systems (3ob) of the Slater−Koster library and 0.005 Ha smearing parameter. The energy tolerance was recorded to be 1 × 10^−1^ kcal/mol throughout geometry optimization.

Before the MD simulation of MIP prepolymerization systems, all studied molecules were energetically minimized using a Condensed-phase Optimized Molecular Potential for Atomistic Simulation Studies (COMPASS) force field [35,36] with 5000 steepest descent and 5000 conjugate gradients to eliminate any bad contacts [37]. To provide a suitable environment for the molecular simulation, five periodic cells were then constructed with the 0.1 g/cm^3^ density and 298 K bias temperature containing all polymerization components for MIP synthesis. As shown in Table 1, the periodic cells were loaded with different molar ratios of MIP components in accordance with the experiment. The all-periodic systems were then optimized under the Smart algorithm and the COMPASS force field to reach their minimum energy configuration.

The MD simulations were conducted in several stages with the use of the COMPASS force field to achieve the accurate and final energy-optimized systems. First, the polymer systems were equilibrated under canonical ensemble (i.e., constant number of particles, volume, and temperature ensemble; NVT) at room temperature (298 K) with a time step of 1.0 fs and a total simulation time of 200 ps. Canonical ensemble allows control over both the volume and temperature during simulation. Afterwards, the MD was carried out by heating up the system to 333 K for 200 ps with canonical ensemble, followed by cooling the systems back to normal temperature (298 K) with the similar dynamics parameter. This step was implemented to allow the prepolymerization system to cross over the energy barriers as well as to reproduce the experimental temperature during polymerization. The Nosé−Hoover thermostat was used throughout the equilibration stage to control the temperature of the NVT simulation. After the optimization and equilibration steps, a simulated annealing process was introduced over all periodic systems with the mid-cycle temperature of 698 K. This step was followed by gradual cooling at room temperature in 10,000 steps under the COMPASS force field and a constant number of particles, volume, and temperature (NPT) ensemble. The Nosé−Hoover thermostat was applied to control the temperature, while the Andersen barostat was used to control the pressure during the simulation. Lastly, the systems were re-equilibrated at 298 K for 200 ps with an NPT ensemble. The Andersen algorithm was applied to control both the temperature and pressure of the system. Radial distribution functions (RDFs), denoted as g(r), were analyzed upon MD simulations to evaluate the structural characteristics of the prepolymerization system and the probabilities of reactive atoms of the functional monomer at various separation distances from the MIP template (i.e., CagA) [38].

### 2.3. Binding Energy Calculation

The binding energy (E_b_) of the prepolymerization systems were calculated as per Equation (1):

(1) $${\;E}_{b}={\;E}_{\mathrm{complex}}-{\;E}_{\mathrm{component}}$$

where E_complex_ is the total energy of the MIP complex and E_component_ is the total energy of the MIP component including the monomer, template, cross-linker, and porogen. Total energy is defined as the sum of valence energy and non-bond energy upon geometry optimization with the Smart algorithm and COMPASS force field. The convergence tolerances for energy, force, stress, and displacement were recorded to be 2 × 10^−5^ kcal/mol, 1 × 10^−3^ kcal/mol/Å, 1 × 10^−3^ GPa, and 1 × 10^−5^ Å.

### 2.4. Synthesis of Nanomaterials

The gold nanoparticles (AuNPs) and rGO were synthesized by using earlier reported protocols. According to the protocol, hydrogen tetrachloroaurate was dissolved in distilled water and kept at 100 °C with continuous stirring. Five ml of 1% trisodium citrate was heated separately and quickly added into a boiling solution of hydrogen tetrachloroaurate. The yellowish color of the solution became transparent and, finally, a wine red color was obtained. The solution was further heated up for 15 more minutes and allowed to cool at room temperature [39]. Furthermore, rGO was synthesized by using a modified Hummer’s method. Two gm of graphite powder was measured and dissolved slowly in 50 mL sulphuric acid (H_2_SO_4_). The flask was kept in an ice bath continuously and left undisturbed for 30 min. After that, 6 gm of KMnO_4_ was slowly added to the above solution. The prepared suspension was stirred continuously for about 2 h and then sonicated. Next, 150 mL of distilled water was slowly added to dilute the sulphuric acid and a further 8 mL H_2_O_2_ was added to stop the reaction. Immediately, the yellow color of the solution was observed. The prepared solution was centrifuged, and the obtained black powder was washed with diluted HCl and distilled water several times. The obtained product was vacuum-dried for 24 h and the final product was dried and stored until further used. Furthermore, to prepare rGO, 0.1 gm graphene oxide powder was dissolved in 150 mL dist. water and sonicated for 2 h. The obtained brown solution continuously stirred in a paraffin oil bath and 1 mL of hydrazine monohydrate was added dropwise very slowly. Again, the solution was kept on the stirrer for 18 h at 90 °C. The color of the mixture changed to black and when the stirring stopped, the hydrophobic particles seemed to settle down at the bottom [40,41].

### 2.5. Synthesis of CagA-Specific MIP

The MIP was synthesized by using a bulk polymerization and electrochemical polymerization method with an optimized ratio of methyl methacrylate (MMA) (monomer), EGDMA (cross-linker), and dilution of CagA analyte. Briefly, monomer, cross-linker, and CagA antigen were mixed together in acetonitrile (ACN) with an initiator Azobisisobutyronitrile (AIBN). This prepared solution was continuously stirred for 12–16 h at 70 °C for bulk polymerization. After the polymerization process, the obtained product was crushed and dried in a vacuum oven overnight to completely remove the moisture. Again, the CagA antigen removal was performed by using methanol and acetic acid in a 4:1 ratio twice and pure methanol once. The end-product was further dried and kept in vacuum-dried condition till further use. The prepared MIP with antigen and without antigen (after washing) were characterized through SEM analysis of the polymer matrix.

### 2.6. Fabrication of Biosensing Surface

#### 2.6.1. Electrodeposition of Nanomaterials on an Electrode Surface

The screen-printed electrodes (SPE) were used for the fabrication of the detection platform. rGO and gold nanomaterials were deposited on SPE using the cyclic voltammetry method. First, the rGO was deposited on the SPE by using a potential range of 0.5 to −1.5 for 6 cycles at a 50 mV/sec scan rate. Again, the gold nanomaterial was deposited through electrodeposition at a 50 mV/s scan rate with a 0.3 to −0.5 potential range for 5 cycles [42,43].

#### 2.6.2. Electropolymerization of CagA-MIP

The rGO and gold nanomaterial-decorated SPE (Au/rGO@SPE) was further modified with CagA-specific MIP. The modification step was performed by electropolymerization (CV) in a potential range of −0.2 to 0.6 V with a scan rate of 50 mV/s for 15 cycles. To remove the CagA template from MIP, the SPE surface was washed with methanol and acetic acid mixture 2 times and only methanol 1 time each for 15 min and dried at room temperature [44].

### 2.7. Electrochemical Response Study

The fabricated sensing platform was studied electrochemically after each modification step to analyze the reaction response on the SPE surface. All the measurements were taken in the presence of electrolytes containing 0.1 M K_3_[Fe(CN)_6_] and K_4_[Fe(CN)_6_] by using a three-electrode cell configuration. The study involved electrochemical measurement through cyclic voltammetry (CV), electrochemical impedance spectroscopy (EIS), and differential pulse voltammetry (DPV) [45].

### 2.8. Study of the Analytical Performance of Fabricated Biosensor

The analytical performance of the fabricated sensing platform was examined with different concentrations of the CagA template. The electrode was also optimized for different pH and temperatures. The effect of different pH (ranging from 4–10) was observed in the presence of 0.1 PBS buffer prepared by the standard method. To optimize temperature for better functionality of the fabricated sensing electrode, various temperatures were applied such as 10, 20, 30, 40 50, 60, and 70 °C. Other than these basic parameters, the selectivity and stability of the developed electrochemical biosensor were assessed by observing the current response in the presence of various interferents like acetylcholine (Ach), ascorbic acid, glucose, uric acid, and BabA and VacA (*H. pylori*-specific other antigens). Furthermore, the stability of the fabricated electrode was studied for up to 60 days by performing DPV once per week under optimum conditions.

### 2.9. Real Sample Analysis

The efficacy of the fabricated sensing platform was observed by electrochemically measuring the analytical performance in the presence of spiked blood samples. For this, blood samples were spiked with known concentrations of the analyte CagA antigen. The prepared spiked blood samples were applied to the working electrode and incubated at room temperature for 10 min. Then, after 10 min, the current response was measured using the CV technique [46].

## 3. Results and Discussion

**Scheme 1:**
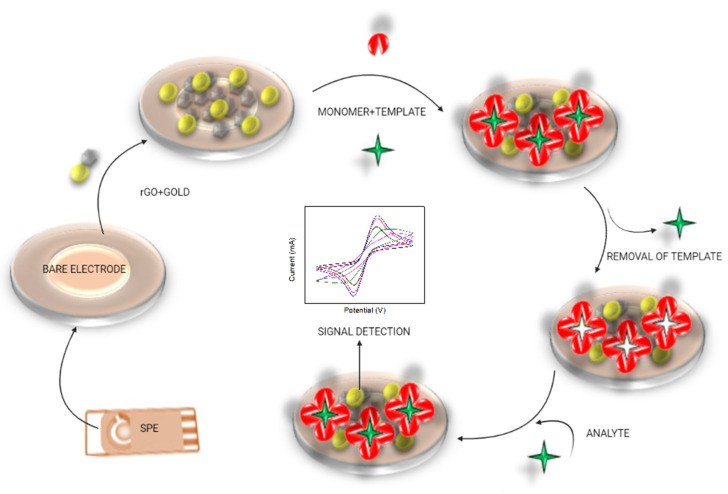
Graphical representation of the fabrication steps involved in the development of the MIP-based biosensor for the detection of the CagA virulence factor of *H. pylori*.

A novel MIP-based electrochemical biosensor was constructed for the detection of the CagA antigen of *H. pylori* bacteria as illustrated in Scheme 1. The deposition of nanoparticles on the electrode surface facilitates the deposition of the biorecognition element by providing a high surface area and a remarkable electrochemical response toward the target analyte.

### 3.1. Morphological Study of Synthesized CagA-MIP by SEM

**Figure 2:**
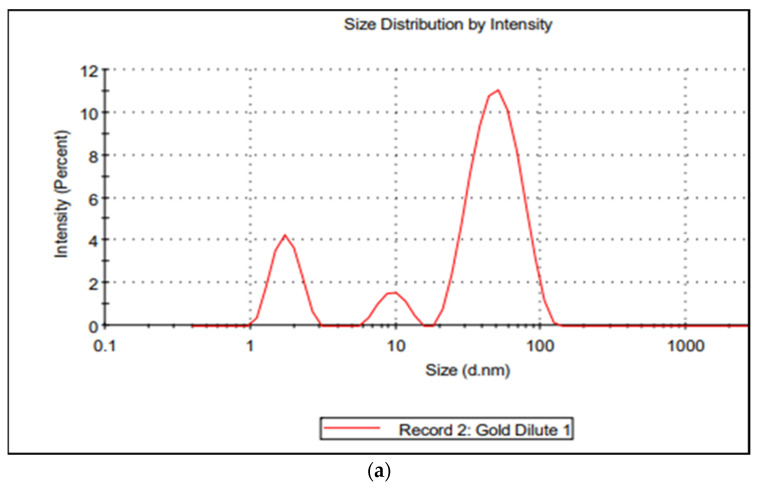

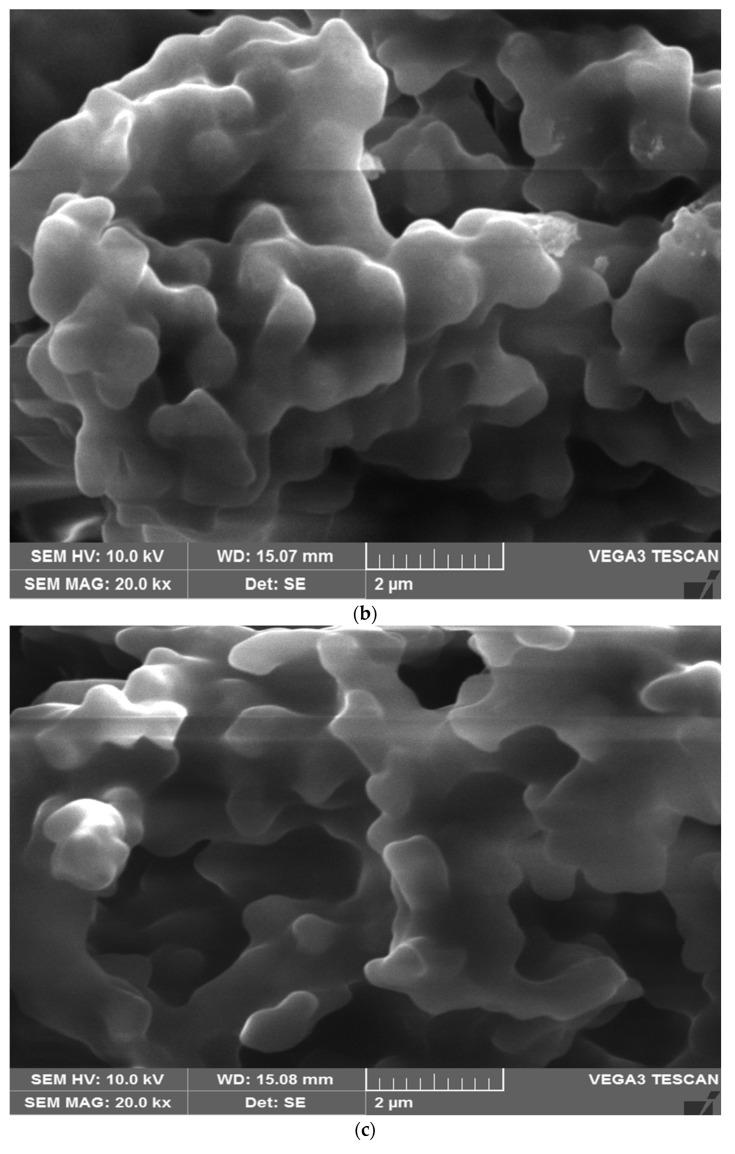

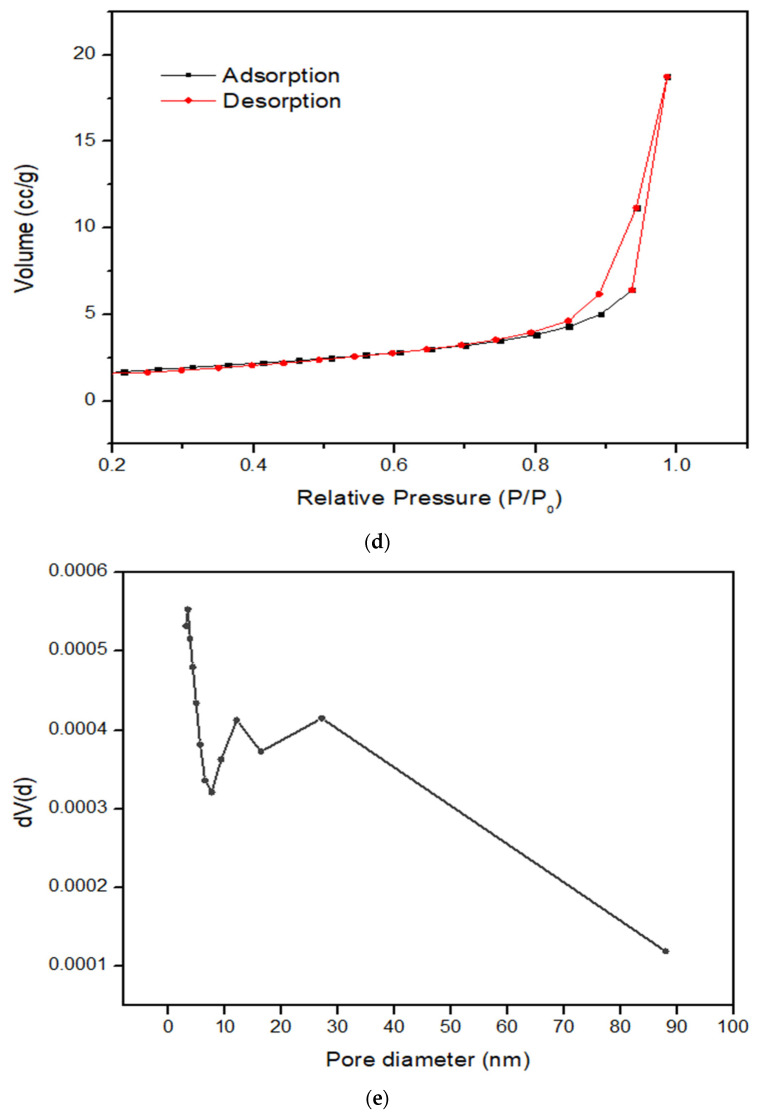
(**a**) Determination of the size of gold nanoparticles via the DLS technique. (**b**) Surface characterization of synthesized MIP with CagA template. (**c**) Surface characterization of synthesized MIP after removal of the CagA template. (**d**) BET isotherm; adsorption and desorption curve. (**e**) Analysis of pore size distribution for MIP by BET.

The size of synthesized gold nanomaterials was observed via the dynamic light scattering (DLS) method. The particles are able to scatter light depending on their diameter to the sixth power. The size of particles measured by DLS is the hydrodynamic diameter of the spheres and is influenced by all the adsorbed substances on the surface of the nanoparticles. Figure 1a indicates that the monodisperse nanoparticles with sizes 60 ± 20 nm are present in colloidal solution. The intensity is highest for larger particles, as can be seen in Figure 2a, whereas the middle peak shows the lowest intensity which may be due to the coagulation of nanoparticles in the sample or the presence of any impurities on the particle’s surface. A possible reason behind this could be the improper sonication of samples to obtain a homogenous solution before measurement.

The synthesized MIP was characterized by SEM to study the surface morphology of the polymer matrix [47,48]. SEM images of unwashed MIP and washed MIP (after removal of CagA antigen) are shown in Figure 2b,c. Figure 2b shows the smooth surface of CagA-MIP before the removal of antigen, which is obviously different from Figure 2c where the rough surface with a large number of holes represents the removal of the template from the polymer matrix. The presence of cavities as shown in Figure 2c indicates the successful imprinting of CagA antigen in the polymer matrix. Pore size distribution and nitrogen adsorption−desorption isotherm of synthesized MIP were observed through BET analysis after removing the template. Figure 2d,e described the adsorption and desorption curve and pore size distribution, respectively. The BET analysis shows the microporous structure of synthesized MIP. Figure 2d depicts the absorption and desorption isotherm of MIP that showed monolayer adsorption. The presence of micropores provides large surface area to access electrode materials very quickly for active species. According to this characterization, the BET surface area of MIP was observed as 5.074 m^2^/g. The pore diameter for most of the MIP’s pores are centered at approximately 10 nm and 28 nm, which showed a bimodal size distribution (Figure 2e). The pore volume and pore diameter obtained for MIP was 0.029 cc/g and 3.374 nm, respectively, as shown in Table 2. From this BET isotherm and pore size distribution analysis, MIP is expected to facilitate electrochemical applications [49,50].

### 3.2. Geometry Optimizations

**Figure 3:**
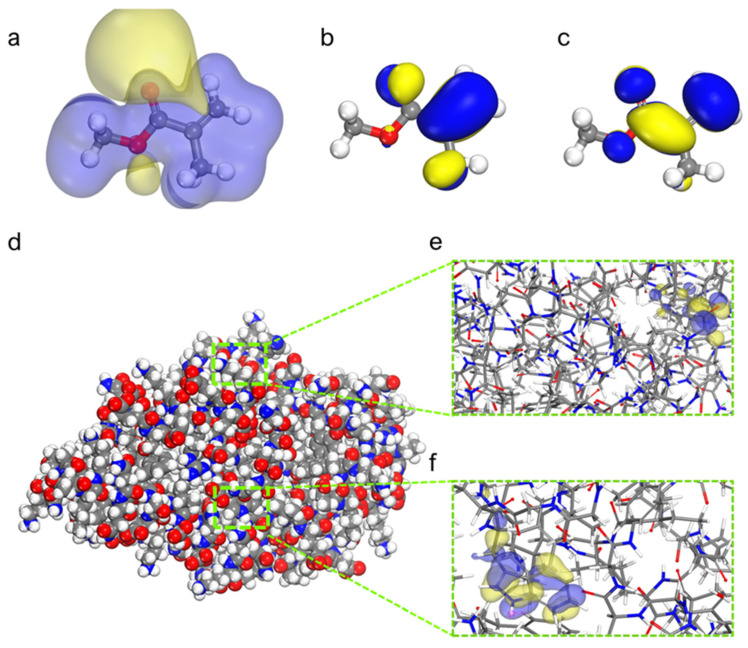
(**a**) Electrostatic potential, (**b**) HOMO, and (**c**) LUMO isosurfaces of MMA after geometry optimization using the B3LYP hybrid functional. (**d**) Optimized structure, (**e**) LUMO, and (**f**) HOMO isosurfaces of CagA after geometry optimization with DFTB+ with 3ob Slater−Koster library.

Computational simulation has been effectively used to elucidate the nano−bio interaction in biosensor platforms [51,52]. In this study, the polymerization process of MIP was optimized through molecular modelling in order to determine the energetic contributions towards the binding event of the MIP template. To realistically simulate the MIP component, the overall molecular structures of MIP were constructed and energetically optimized. In the first step, the electrostatic potential (ESPs) and the highest occupied molecular orbital (HOMO)−lowest unoccupied molecular orbital (LUMO) isosurfaces were analyzed via B3LYP-based DFT optimizations to observe the electron localization and reactive sites of the functional monomer. The ESPs display the region of the electric field where yellow- and blue-colored areas represent negative and positive potentials, respectively [53]. As seen in Figure 3a, the electron cloud is mostly accumulated around the O atoms of the MMA due to their high electronegativities as compared to the C-H backbone, which tends to be the electropositive area. ESPs enunciate the reactive site of the MMA, which is predominantly driven by electronegative O atoms. These two O atoms in the monomer could form recognition imprinting sites toward the template molecule during MIP synthesis. Frontier orbital analysis was further studied to assess the chemical reactivity of the MMA (Figure 2b,c). It is also well-known that HOMO−LUMO plots depict the electronic density and, therefore, demonstrate the accumulation of reactive sites in molecules [54]. As a result, HOMO−LUMO lobes were mainly delocalized at the O atoms and neighboring C backbone of the monomer, with a small contribution coming from the atoms which are away from the electronegative oxygens. This result emphasizes the chemical reactivity of the MMA functional monomer, which is predominantly derived from the O atoms, in agreement with the ESP analysis.

Furthermore, the optimized structure and frontier orbital isosurfaces of the CagA template is depicted in Figure 3d–f. After the DFTB+-based geometry optimization, the HOMO isosurface was primarily distributed in tryptophan (Trp) amino acid, while the LUMO contour was delocalized in phenylalanine (Phe). The results show that the main reactivity of the CagA was driven by two aromatic amino acids in the protein building block due to the resonance delocalization of π-electrons in the benzene ring of Phe and indole ring of Trp [55,56]. However, due to the lack of hydrogen donor in the Phe side chain, only Trp was included in the RDF observation in subsequent analyses.

### 3.3. Molecular Dynamics Simulations and Binding Energy Calculation

**Figure 4:**
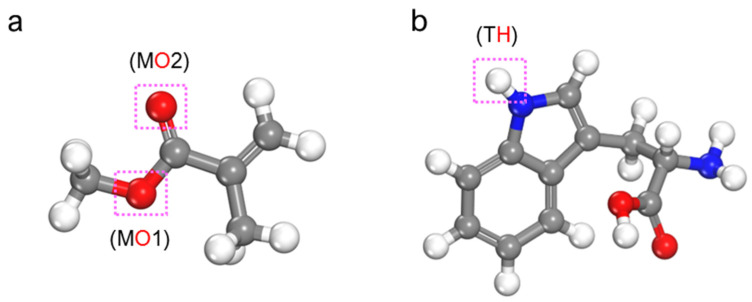
Chemical structures of (**a**) MMA and (**b**) tryptophan of CagA along with their labelled atoms for RDF analysis.

The proximity of the CagA to MMA degrees are discussed in this section from the RDF standpoint after the MD treatments. Figure 4 shows the labeled atoms at the MMA monomer and Trp of CagA that could form recognition imprinting sites on the basis of ESPs and frontier orbital analysis from DFT calculations [37,57,58]. In MMA, two oxygen atoms (i.e., MO1 and MO2) were assigned as the possible hydrogen bonding acceptors (Figure 4a), while the TH at Trp was assigned as the proton donor for the RDF analysis (Figure 4b). In general, RDF is essential to the statistical mechanics and demonstrates the probability of finding a particle in an infinitesimal shell at a distance r from another particle in the liquid state [59]. RDF is useful to predict the conditions of the MMA molecules around the CagA template [37].

**Figure 5:**
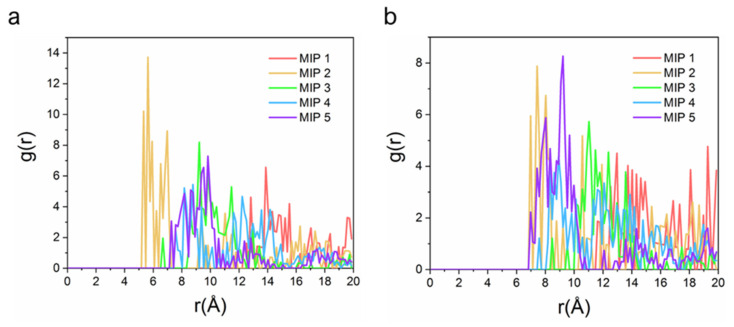
RDFs of (**a**) TH−MO1 and (**b**) TH−MO2. g(r) shows the RDF values, while r(Å) represents the distance from a solute atom and the average bulk atom.

As a result, TH (atom H labeled in Trp)−MO1 (atom O1 labeled in MMA) atomic contact appeared (Figure 5a). Among other MIP optimization mixtures, MIP 2 shows the lowest radius (distance) to TH, indicating a stronger molecular interaction between functional monomers and CagA. Due to the 5–6 Å radius recorded to TH, the TH, the TH−MO1 in the MIP 2 interaction may be dominated by weak hydrogen bonds. The highest RDF was observed in MIP 2 at 13.7 of g(r) peak with 5.62 Å of r, which demonstrates a greater possibility of finding MMA at a closer atomic distance to the CagA template. Meanwhile, the RDF of the TH−MO2 is depicted in Figure 5b. Herein, both MIP 2 and MIP 5 showed very close radii to TH with a higher g(r) peak observed in MIP 2 than in MIP 5 at a closer distance to Trp. In terms of MO1 and MO2 interactions in MIP 2, MO1 is predicted to be the main interaction site of monomer with CagA due to its comparatively stronger action site than MO2. These interactions may belong to weak hydrogen bonding rather than strong hydrogen bonding at a short distance (~2.7–3.0 Å) [60,61]. Thus, from the RDF point of view, the adsorption capacity of MIP 2 could be greater than other studied MIP mixtures shown in Table 1. This finding is in good agreement with the experimental molar ratio during MIP synthesis.

Binding energy was further characterized to elucidate the interaction energy of the MIP complex, particularly before and after template removal. It is well-known that template removal is a critical step in the synthesis to form an imprinted site which is responsible for the recognition properties of the MIP [62]. The E_b_ was calculated according to Equation (1) from the MIP 2 as the optimized mixture of MIP preparation (Table 3). As a result, E_b_ before the template removal was significantly lower than after template removal, indicating a high interaction strength between the receptor (MIP) and target (CagA). This could lead to the high sensitivity of the biosensor for the recognition of the specific target. After the formation of the imprinted site, it is well expected that E_b_ remains higher due to the absence of stable CagA protein in the complex system. As seen in Table 3, CagA possesses a remarkably low total energy than other molecules (i.e., −6878.95 kcal/mol). Indeed, a low E_b_ upon template removal indicates the capability of MIP to recognize the biosensor target (CagA) through a robust binding mode and affinity.

### 3.4. Electrochemical Experiment Results

#### 3.4.1. Validation of Fabrication Steps through Electrochemical Study

**Figure 6:**
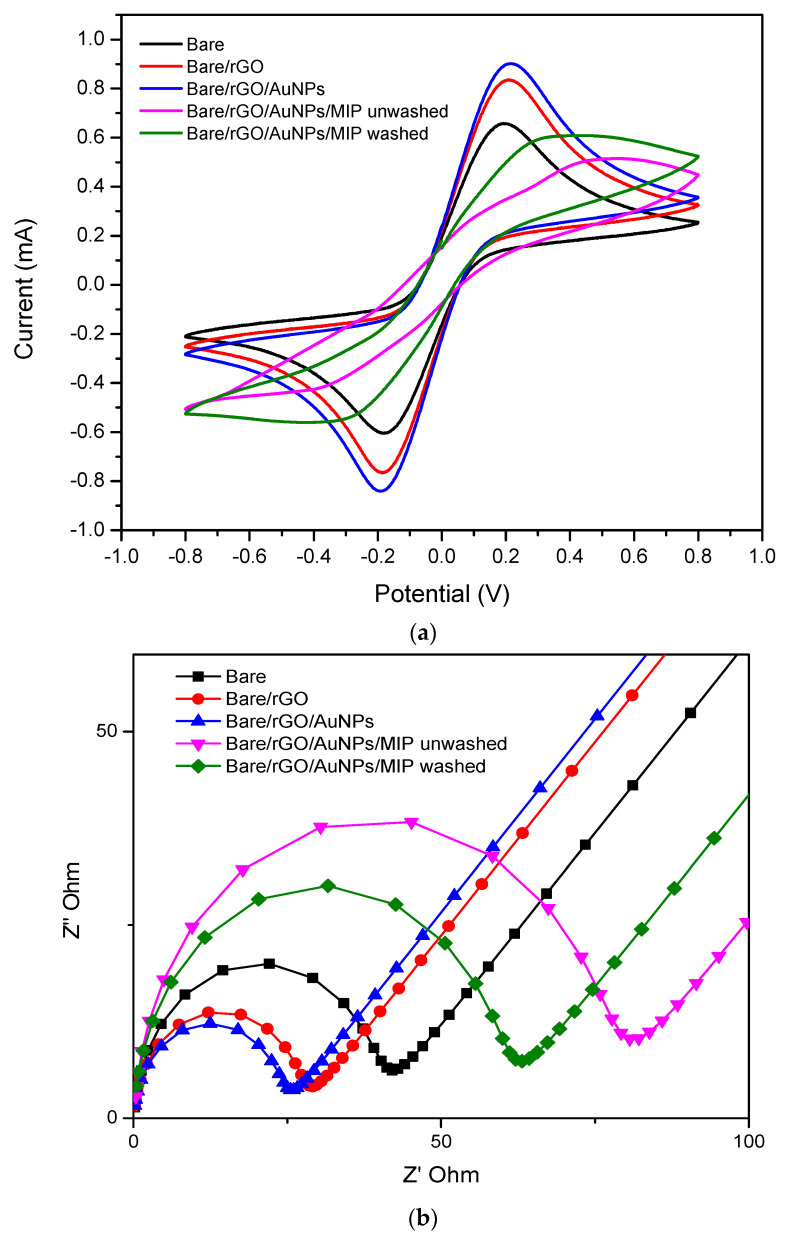
(**a**) Electrochemical response studies via the CV technique for Bare SPE (black), rGO@SPE (red), AuNPs/rGO@SPE (blue), MIP unwashed/AuNPs/rGO@SPE (green), and MIP washed/AuNPs/rGO@SPE (magenta) obtained in the presence of [Fe(CN)_6_]^3−/4−^ electrolyte at 50 mV/s. (**b**) The EIS study of Bare SPE (black), rGO@SPE (red), AuNPs/rGO@SPE (blue), MIP unwashed/AuNPs/rGO@SPE (magenta), and MIP washed/AuNPs/rGO@SPE (green) at the frequency range of 10–2000 Hz.

The electrochemical behavior of the modified electrode was investigated at each step by cyclic voltammetry and electrochemical impedance study (EIS) curves. Performance was determined through a comparison of CV signals after each deposition step. Figure 6a shows the CV curves in a potential range of −0.8 to 0.8 V, belonging to the bare electrode, rGO and gold NP-coated electrodes, MIP-modified electrode, and the electrode modified by MIP after the removal of the template in 0.1 M ferro/ferri solution. After modification with rGO and gold NPs, the current intensity was increased in comparison to bare SPE due to the high conductivity and extended surface area of rGO and gold. Further modification of SPE by MIP decreased the current intensity, which indicates the large transfer resistance of polymer-modified SPE. This could be attributed to the large electron transfer resistance of MIPs. After the removal of the template, the current increased significantly due to the formation of CagA-specific imprinting cavities that resulted in the enhancement of electrolyte diffusion and the acceleration of electron transfer on the SPE surface. The same steps were validated through EIS and also showed the resistance developed at each step in Figure 6b [63].

#### 3.4.2. Optimization of Fabricated Electrodes for Various Sensing Parameters

**Figure 7:**
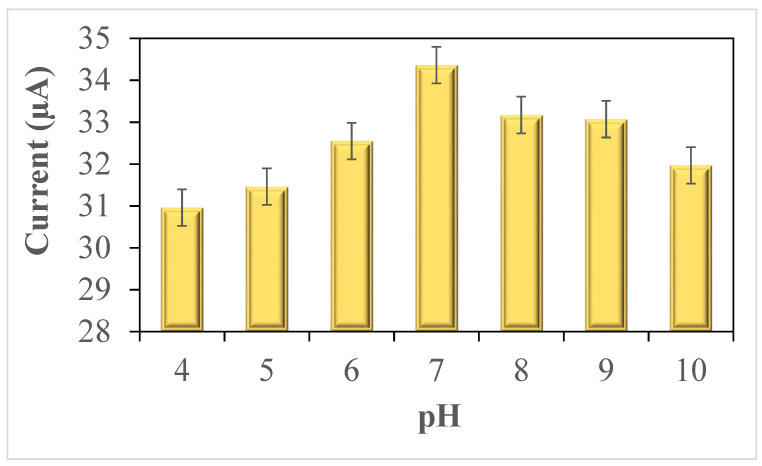
Effect of various pH on the analytical performance of the MIP-based electrochemical biosensor.

**Figure 8:**
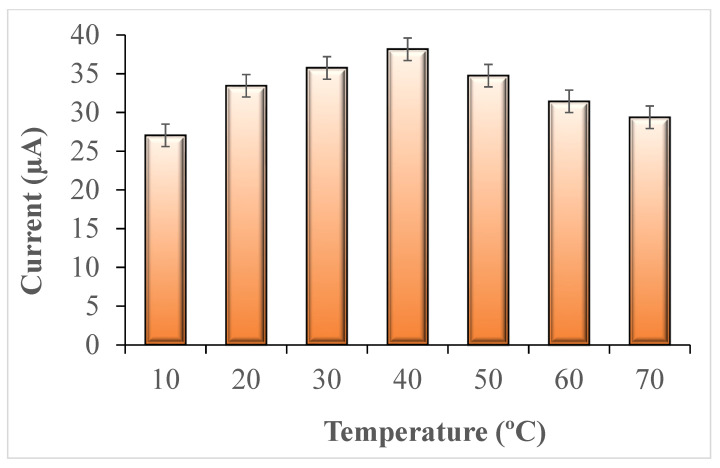
Determination of optimum temperature for the fabricated sensing platform.

To achieve the optimum sensing conditions like pH and temperature, the fabricated electrode was examined at various ranges. The influence on sensing performance due to the effect of various pH values is illustrated in Figure 7. The increase in the pH value from 4 to 10 reflected the improvement in electrode performance. However, after 7, the current intensity decreased to the pH range of 8 to 10, which indicates the binding of MIPs was prevented beyond 7 pH. Thus, the optimum pH was observed as 7 based on which MIPs showed remarkable recognition performance. The fabricated sensing platform was also examined with varying temperatures in the range of 10–70 °C. The bar graph was plotted against the different DPV currents at various temperatures (Figure 8). The DPV current was increased from 10- 40 °C and then dramatically decreased beyond 40 °C. Thus, we selected 40 °C as the optimum temperature for this fabricated electrode.

#### 3.4.3. Sensor’s Analytical Performance

**Figure 9:**
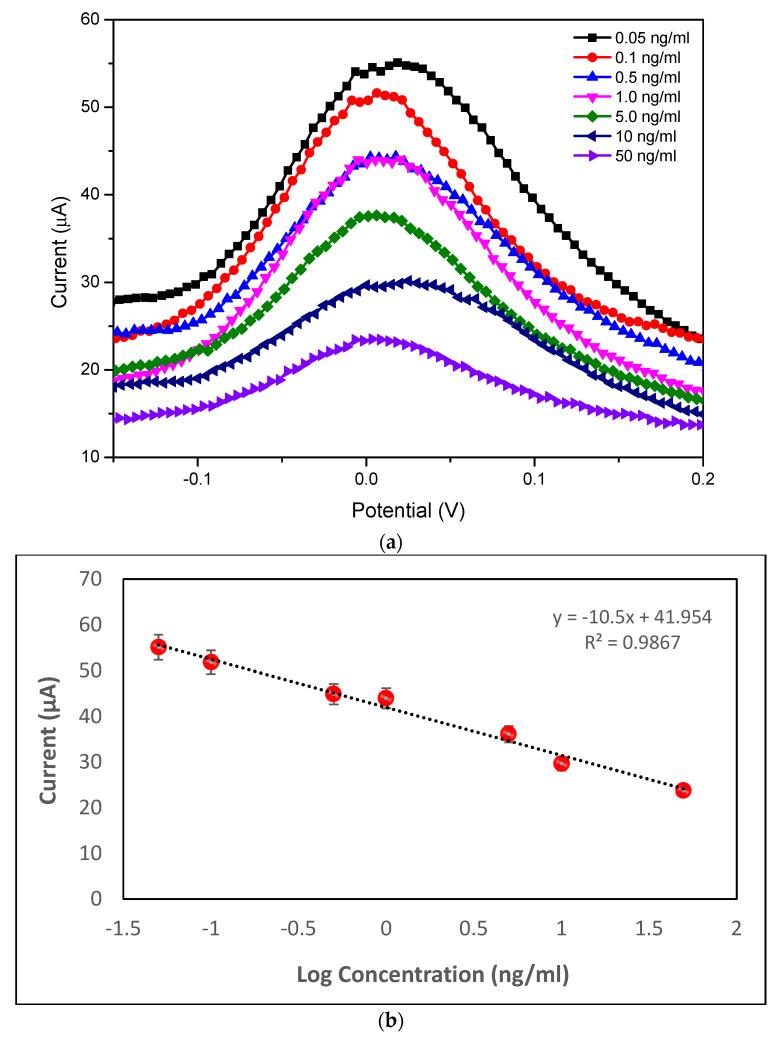
(**a**) DPV response study of MIP-CagA/Au/rGO@SPE with different concentrations of CagA antigen in the presence of [Fe(CN)_6_]^3−/4−^ electrolytes. (**b**) Representation of the standard calibration curve of MIP-CagA/Au/rGO@SPE with different concentrations of target analytes with reference to DPV results.

The electrochemical response of the fabricated electrode CagA-MIPs/Au/rGO@SPE was further investigated by using various concentrations of CagA. The differential pulse voltammetry (DPV) was used to record current responses of various concentrations (Figure 9a). The concentration study was performed within a concentration range of 0.05 ng/mL to 50 ng mL^−1^ (0.05 ng mL^−1^, 0.1 ng mL^−1^, 0.5 ng mL^−1^, 1 ng mL^−1^, 5 ng mL^−1^, 10 ng mL^−1^, 50 ng mL^−1^). The concentration study was carried out in ferro/ferri buffer solution [64]. The DPV graphs show that the magnitude of peak current response decreases significantly with an increase in CagA concentration. The acquired result depicts that at higher concentrations of CagA, active sites on the surface of the electrode are blocked due to greater occupancy of cavities, which is attributed to the blocking of electron transfer via buffer solution. Thus, the low current is generated at the highest provided (50 ng mL^−1^) concentration. The calibration graph is also plotted between the current response and different concentrations, as shown in Figure 9b. The graph shows a linear curve with high sensitivity as 0.325 µA ng^−1^ mL^−1^. The limit of detection was reported as 0.05 ng mL^−1^.

#### 3.4.4. Scan Rates Study to Observe Sensing Response

**Figure 10:**
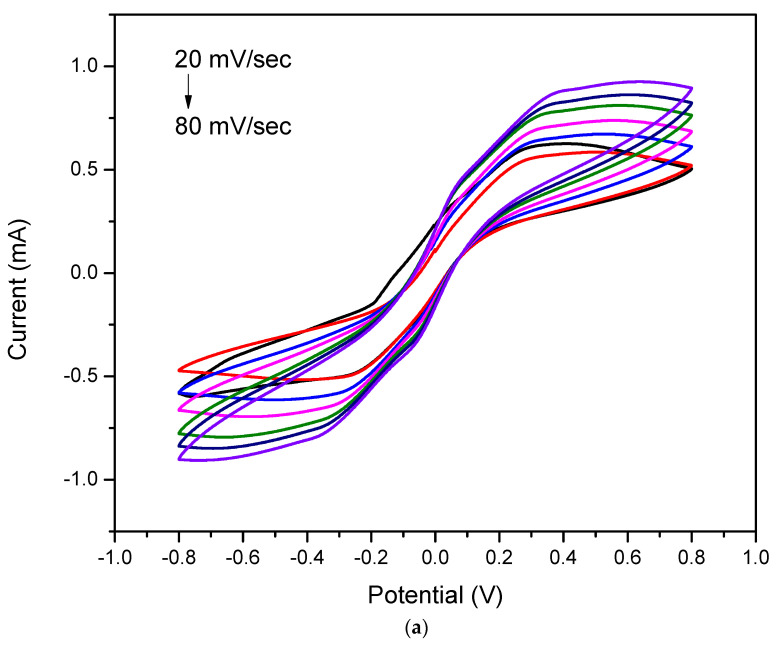

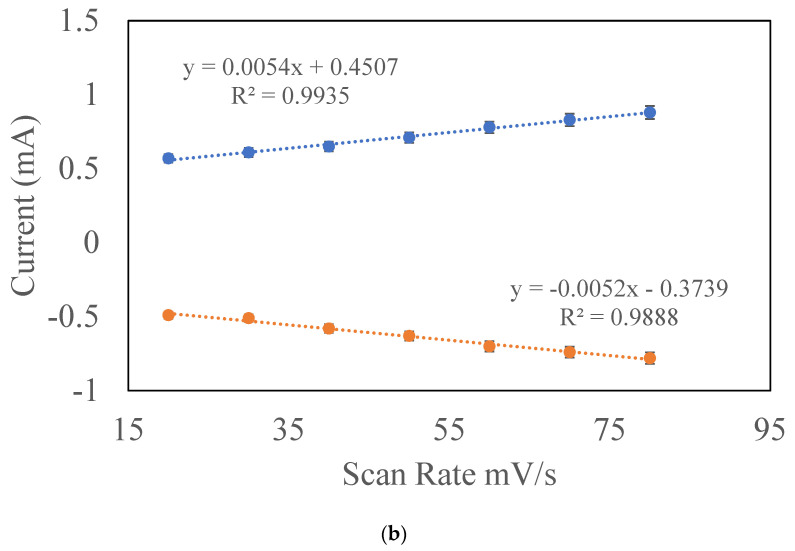
(**a**) CV recorded for MIP-CagA/Au/rGO@SPE at different scan rates in the presence of [Fe(CN)_6_]^3−/4−^ electrolytes. (**b**) Representation of variations in the peak oxidation current (Ipa) and reduction peak current (Ipc) at different scan rates than the linear graph.

In Figure 10a, the CV study demonstrates the biosensing response of the modified electrode at different scan rates, i.e., from 20–80 mV/s. The voltage for scan rate study was applied from +0.8 to −0.8 V. This study stated a ‘quasi-reversible’ process to observe electron transfer by diffusion reaction. The CV plot shows the symmetrical reduction and oxidation peaks that depict the relatively equivalent potential response. With the increase in the scan rate range, the peak currents also increased, which shows the steady electron transfer kinetics on the modified electrode surface. In Figure 10b, the square root of the scan rates has been demonstrated which is observed from 20–80 mV/s. The scan rate is equivalent to the redox peak current. The relationship between the current response and the square root of the scan rate has been plotted as follows:

y = 0.0054x + 0.4507; R^2^ = 0.9935 (2)

y = −0.0052x − 0.3739; R^2^ = 0.9888 (3)

The oxidation, as well as the reduction current peaks, are linearly relative to the relevant scan rate’s square root.

#### 3.4.5. Analysis of Selectivity and Stability

**Figure 11:**
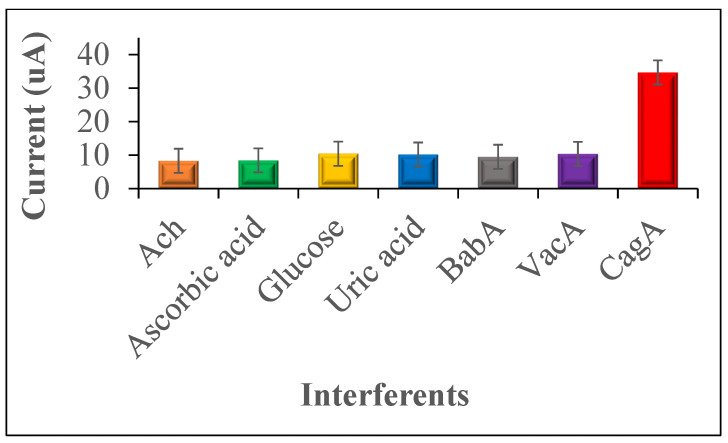
Plot of interferent studies of MIP-CagA/Au/rGO@SPE with 0.05 ng/mL CagA antigens.

**Figure 12:**
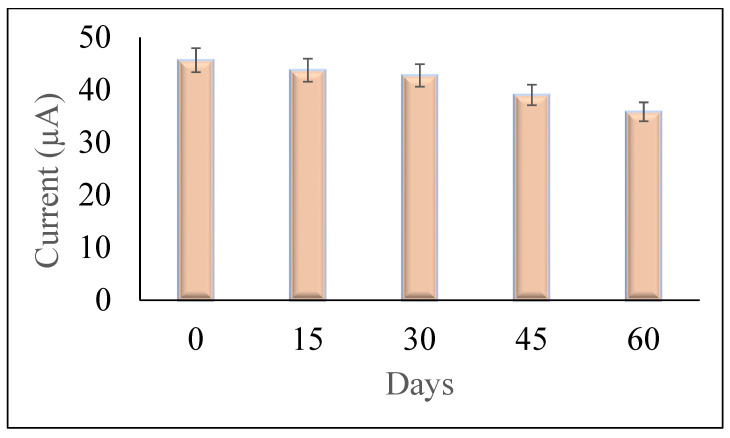
Demonstration of the fabricated sensing electrode’s stability over the period of 60 days.

The interference of various biomolecules like glucose, cholesterol, ascorbic acid, *H. pylori*-specific BabA, and VacA was studied for modified electrode. For clinical use, a sensor must show high specificity for the target analyte in comparison to other interfering biomarkers that are present in the blood serum. As shown in Figure 11, the magnitude of the DPV current was observed as 34.68 μA for CagA-MIP/Au/rGO@SPE. After that, the DPV’s current response was taken in the presence of the various abovementioned interferents. Although there were slight variations in the current response, there was no major alteration observed in the current’s response to other interferents as compared to blank. However, after the addition of CagA analyte, a significant increase in the current response was observed. The interference study shows that the fabricated electrode is highly specific for the CagA virulence factor of *H. pylori*. The retained activity of the fabricated biosensor MIP-CagA/Au/rGO@SPE was analyzed in the presence of 0.05 ng/mL of CagA antigen to observe current response incubating in a mixture at regular intervals (every week). The fabricated electrode was stored at 4 °C and observed for up to 60 days for any reduction in its performance. The fabricated sensing platform retained its original activity by approximately 80% over the period of 60 days (Figure 12).

#### 3.4.6. Real Sample Analysis

Further, the fabricated MIP-based sensing platform was observed for clinical applications by investigating it with spiked blood samples. Results showed that the developed biosensor is very selective for the diagnosis of *H. pylori* and easy to handle. CagA antigen was analyzed in the spiked sample and RSD (relative standard deviation) was calculated to determine the precision of the biosensor. Additionally, the recovery percentage was also calculated by using the standard method in the presence of 0.05 ng/mL CagA antigen. The entire experiment was repeated five times to obtain the average recovery in similar conditions. The results showed approximately 96% recovery, which shows the efficacy of the fabricated biosensor for its clinical use in the future.

## 4. Conclusions

Herein, we have developed a highly sensitive rGO and gold NP-based MIP sensor for the detection of the CagA antigen of *H. pylori*. An electrodeposition technique (CV) was used for the deposition of nanomaterials, followed by the electropolymerization of MIP on a screen-printed electrode. The reactive site of functional monomers and the CagA template were elucidated by ESP and frontier orbital analysis, resulting in the potential atomic contacts that are responsible for the formation of imprinted sites. From the RDF standpoint, the adsorption capacity of MIP 2 with CagA as a template, MMA as a functional monomer, and EGDMA as a cross-linker at a ratio of 1:4:4 was greater than other simulated MIP mixtures which matched well to the experimental molar ratio during MIP synthesis. Further, a low E_b_ upon template removal suggests the ability of as-synthesized MIP to recognize the biosensor target through a robust binding affinity. The sensitivity of the fabricated electrode was calculated as 0.275 µA ng^−1^ mL^−1^ with a limit of detection of 0.05 ng/mL. The CagA antigen is the major virulence factor responsible for the establishment of *H. pylori* infection in the human stomach. Thus, the proposed MIP-based biosensor can be developed as a point-of-care device in the future for the detection of gastric cancer in its early stage.

## Figures and Tables

**Table 1:** Prepolymerization mixtures for the molecular simulation of MIP.

Polymer	CagA (mmol)	MMA (mmol)	EGDMA (mmol)	ACN (mmol)	Molar Ratio
MIP 1	1	2	2	80	1:2:2
MIP 2	1	4	4	80	1:4:4
MIP 3	1	6	6	80	1:6:6
MIP 4	1	8	8	80	1:8:8
MIP 5	1	10	10	80	1:10:10

**Table 2:** Parameters for synthesized MIP.

Surface Area	5.074 m^2^/g
Pore Volume	0.029 cc/g
Pore Diameter Dv(d)	3.374 nm

**Table 3:** Total energy and binding energy of the MIP prepolymerization system.

Complex/Molecules	Total Energy (kcal/mol)	Binding Energy (kcal/mol)
Before template removal (CagA-MMA-EGDMA-ACN)	−9049.77	−886.48
After template removal (MMA-EGDMA-ACN)	−1317.29	−32.94
CagA	−6878.95	
MMA	−53.32	
EGDMA	−153.70	
ACN	−1077.32	
